# Supplementary material for: Structural-disorder-driven critical quantum fluctuation and localization in two-dimensional semiconductors
Source: Nat Commun. 2023 Apr 21;14:2283. doi: 10.1038/s41467-023-38024-4 (PMC10121577; doi:10.1038/s41467-023-38024-4)
Supplement: Supplementary file 1 — Supplementary Information [file 41467_2023_38024_MOESM1_ESM.pdf]

Supplementary Information

Structural-disorder-driven critical quantum fluctuation and localization in two-dimensional semiconductors

Bong Gyu Shin[1,2,3], Ji-Hoon Park[4], Jz-Yuan Juo[1], Jing Kong[4], Soon Jung Jung[1*]

[1]Max Planck Institute for Solid State Research; Heisenbergstrasse 1, 70569 Stuttgart, Germany.

[2]SKKU Advanced Institute of Nanotechnology (SAINT), Sungkyunkwan University (SKKU), Suwon 16419, Republic of Korea.

[3]Department of Nano Science and Technology, Sungkyunkwan University (SKKU), Suwon 16419, Republic of Korea

[4]Department of Electrical Engineering and Computer Science, Massachusetts Institute of Technology; Cambridge, Massachusetts 02139, United States.

*e-mail: s.jung@fkf.mpg.de

Supplementary Discussion

Definitions of chemical potential, electrochemical potential, Fermi energy, and Fermi level

The total chemical potential is described using various terminologies depending on the necessity or convention in a system or field. The total chemical potential consists of the chemical potentials of each species or for a species. The total chemical potential of electrons at finite temperatures is often called the Fermi level in a semiconductor system. In an electrochemical or solid-state system, the total chemical potential of the electron is often called the electrochemical potential with definitions of the (internal) chemical potential and electric potential[1]. In this case, the definition of the chemical potential neglects the electrostatic contributions.

Here, the terminologies used in the manuscript are clarified. In the manuscript, a total chemical potential of an electron is called a Fermi level or an electrochemical potential, which have the same meaning. The Fermi energy was used to indicate the Fermi level or the electrochemical potential at 0 K. The usage of the electrochemical potential distinguishes the chemical potential and electrostatic potential as described above. The electrochemical potential is the sum of the chemical potential and the electrostatic potential. In the manuscript, the local band edge fluctuations are responsible for both the fluctuations of the local chemical potential and the electrostatic potential caused by charge localizations. At equilibrium, the electrochemical potential is constant over the system. The local band bending compensates the changes of chemical potential induced by the charge localization and keeps the electrochemical potential constant across the system. Particularly in 2D semiconductors, in contrast to the rigid 3D bulk systems, the local band bending, and the internal chemical potential are very sensitive to the charge localization, owning to the smaller dielectric screening of the Coulomb interactions between charge carriers and the large fluctuation of band gap in the structural disorder.

Relationship between the strain, band gap, and potential

The bending strain energy (E_b) of a curvature (κ) is given by $E_b = (1/2)C_b\,\kappa^2$, where C_b is the bending modulus. The bending strain (ε_b) is proportional to the curvature, $\varepsilon_b = (\mathrm{t}/2)\,\kappa$, where t is the thickness of a monolayer. From the relations, the bending strain energy is proportional to the square of the bending strain, $E_b \propto \varepsilon_b^2$. We assume that strain-induced potential change (ε_p) at a site is due to the bending strain, such that $\varepsilon_p \propto \varepsilon_b$, leading to $E_b \propto \varepsilon_p^2$ (ref.[2,3]). The strain-induced potential change acts like a potential depth of an electron or hole. From the ref.[4], we assume that the curvature-induced band gap change (ΔE_G) is proportional to the bending strain, $\Delta E_G \propto \varepsilon_b$. Therefore, a linear relationship between band gap change and potential depth for the trap is expected, $\varepsilon_p \propto \Delta E_G$ acting like a 'charge basket' in the structural disorder. The local band gap (E_G) is given by the energy gap between VBM and CBM at a position of $\boldsymbol{r}$, $E_G(\boldsymbol{r}) = CBM(\boldsymbol{r}) - VBM(\boldsymbol{r})$, leading to $\varepsilon_p(\boldsymbol{r}) \propto \Delta CBM(\boldsymbol{r})$ for electrons ($\varepsilon_p(\boldsymbol{r}) \propto \Delta VBM(\boldsymbol{r})$ for holes) where $\Delta CBM(\boldsymbol{r})$ ($\Delta VBM(\boldsymbol{r})$) is the local conduction (valence) band edge fluctuation compared to that in the unstrained case.

On the other hand, the depth of the trap with binding energy (E) of the localized states shows a general relationship given by $|E| \approx C_1\,\varepsilon_p^2 - C_2 = C_3\,\Delta E_G^2 - C_4$, where the coefficients of C_1, C_2, C_3, and C_4 depend on the details of the trap and reference potential[2,3]. The relationship with a Gaussian distribution of the band gap fluctuation ($\sim\exp(-\Delta E_G^2/2\sigma_g^2)$, Supplementary Fig. 8) directly suggests the exponential band tails in the density of states, $\sim\exp(-|E|/\varphi_c)$ with $\varphi_c = 2\,C_3\,\sigma_g^2$, where σ_g^2 is the variance of the band gap fluctuation and φ_c is the characteristic width of the band tail[5,6]. As the height fluctuation is larger, upper limits of band tail width and the variance of

band gap fluctuation are expected under the elastic limit of the concerning monolayer. Indeed, the tight-binding results in Fig. 4j show a saturation trend of band tail width as the variance of band gap fluctuation increases. We obtained the best fitting result of $\varphi_c \propto \sigma_g^p$ with p = 0.11 above the σ_g of ~0.08 eV in Fig. 4j.

Intrinsic magnetization and magnetic field dependence of the critical exponent *ν*

From the ref.[7], the curvature-induced pseudo-magnetic field ($\mathcal{B}(\boldsymbol{r})$) at a position ($\boldsymbol{r}$) of the unit-cell in the Monge's representation was given by

$$\mathcal{B}(\boldsymbol{r}) = \pm\frac{\hbar}{2|e|}\kappa(\boldsymbol{r}) \qquad (8)$$

where $\hbar$ is the reduced Planck constant, $\kappa(\boldsymbol{r})$ is the Gaussian curvature, and the sign +(−) indicates up(down)-spin electrons with respect to the local quantization axis introduced by the spin-orbit coupling under the criterion of $\nabla^2 h_s \ll \frac{\Delta_{SO}}{\hbar v_F}$, where h_s is the surface height fluctuation, Δ_{SO} is the spin-orbit coupling strength, and v_F is the Fermi velocity of charge carriers. We obtained the average values of the Gaussian curvature ($\langle\kappa\rangle$) from STM images and achieved the average value of the pseudo-magnetic field ($\langle\mathcal{B}\rangle$) from the eq. (8). The average values of the Gaussian curvatures from different areas of STM results show the same order of numerical values as a typical value.

The averaged magnetization ($\langle M\rangle$) of monolayer MoS_2 from the pseudo-magnetic field was calculated by $\langle M\rangle = (\chi_{MoS_2}/\mu_0)\langle\mathcal{B}\rangle$ for $\chi_{MoS_2} \ll 1$, where μ_0 is the vacuum permeability and χ_{MoS_2} is the orbital magnetic susceptibility of MoS_2 near the conduction band edge[8].

From the result of ref.[9], the critical exponent ν for the disordered system with random spin-orbit coupling under the weak magnetic field (B) was given by

$$\nu(B) = \nu_0 + \frac{0.6B^{0.25}}{\sqrt{B_c - B}} \times \frac{1}{1 + 0.02(B_c - B)^{\mu}\exp\left(\frac{0.6B^{0.25}}{(1-\gamma)\sqrt{B_c - B}}\right)} \qquad (9)$$

where ν_0 is the value of 2.73 at the zero magnetic field corresponding to the symplectic case, B_c is the critical value of B, μ is an arbitrary constant, and γ is a constant related the estimation of the critical energy ($\overline{E_c}(B)$) along with the presence of B_c under possible numerical errors, $\overline{E_c}(B) = E_c(B) + \gamma[E_c(B_c) - E_c(B)]$. We reproduced the numerical result of $\nu(B)$ in ref.[9] as shown in Supplementary Fig. 15, using eq. (9). Note that B in eq. (9) is counted in the unit of half of the magnetic flux quantum, $h/|e|$, where h is the Planck constant. The numerical result with $\langle\mathcal{B}\rangle$ = ~0.021 T shows ~7.19 in good agreement with fitting result of ~7.28 from the contour of autocorrelation in Fig. 4e.

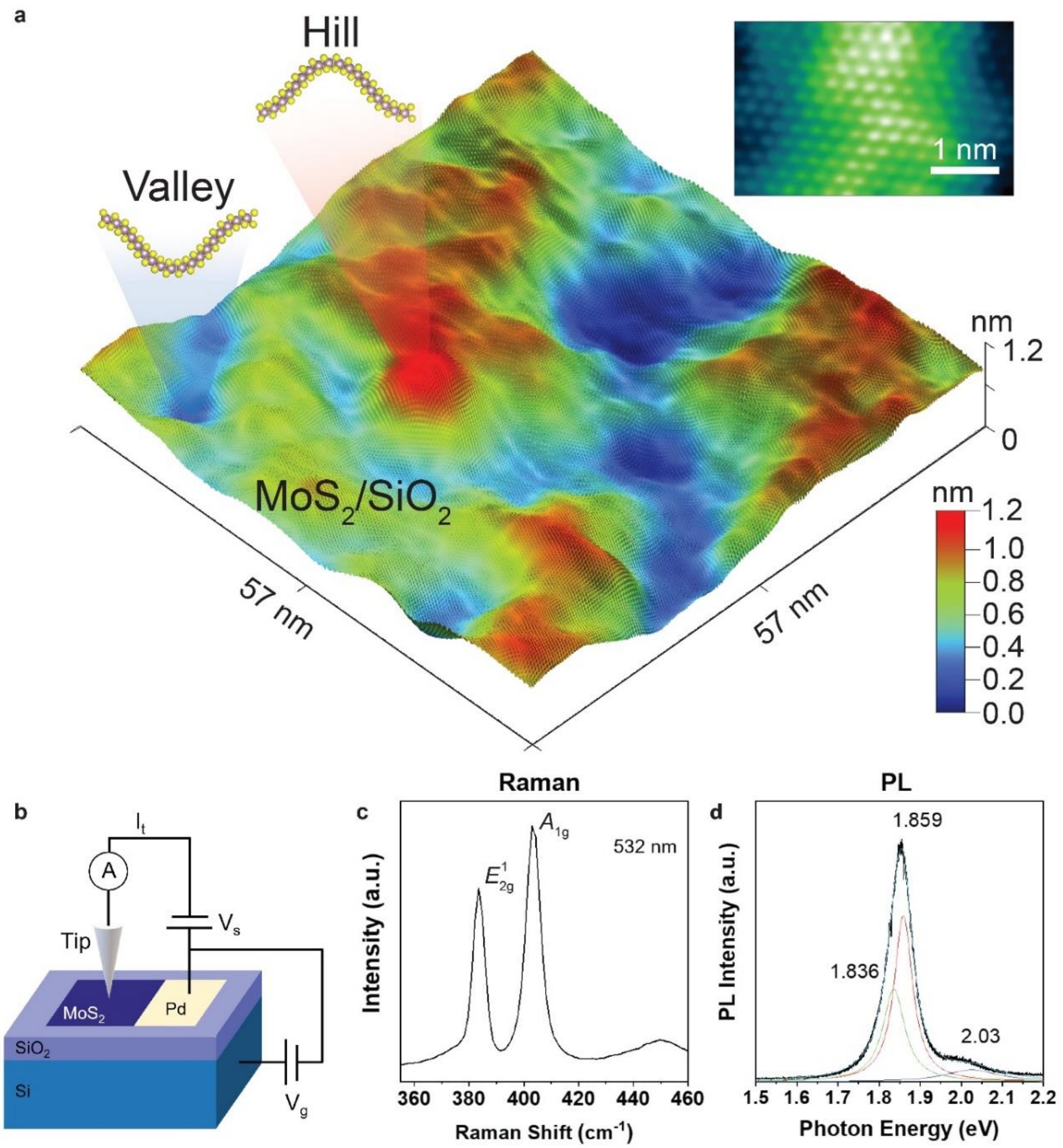

Supplementary Fig. 1 | A representative surface morphology of monolayer MoS_2 on SiO_2 and characterization. **a**, STM image of monolayer MoS_2 on SiO_2 shows randomly deformed structures with valleys and hills over a nanometer-scale. Inset is a close-up STM image exhibiting sulfur-induced protrusions with the honeycomb structure (sample bias $V_s = -3.5$ V and tunneling current $I_t = 1$ nA). **b**, Gate-STM configuration. The sample bias was applied through Pd electrodes on MoS_2. The Pd electrodes are inert and barely oxidized under ambient conditions. The Pd electrodes were less strained on MoS_2 at low temperatures due to the small difference in thermal expansion coefficients between Pd and MoS_2. **c**, Raman spectroscopy shows peak positions of E^1_{2g} (383.5 cm^{-1}) and A_{1g} (403.5 cm^{-1}) with the feature of the monolayer MoS_2, $E^1_{2g} - A_{1g} = 20$ cm^{-1} (ref.[10]). (Excitation wavelength of 532 nm and a laser power of 10 mW) **d**, Photoluminescence characterized the monolayer MoS_2 with two exciton transitions (red line, 1.859 eV; blue line, 2.03 eV) and a trion emission (green line, 1.836 eV)[11,12].

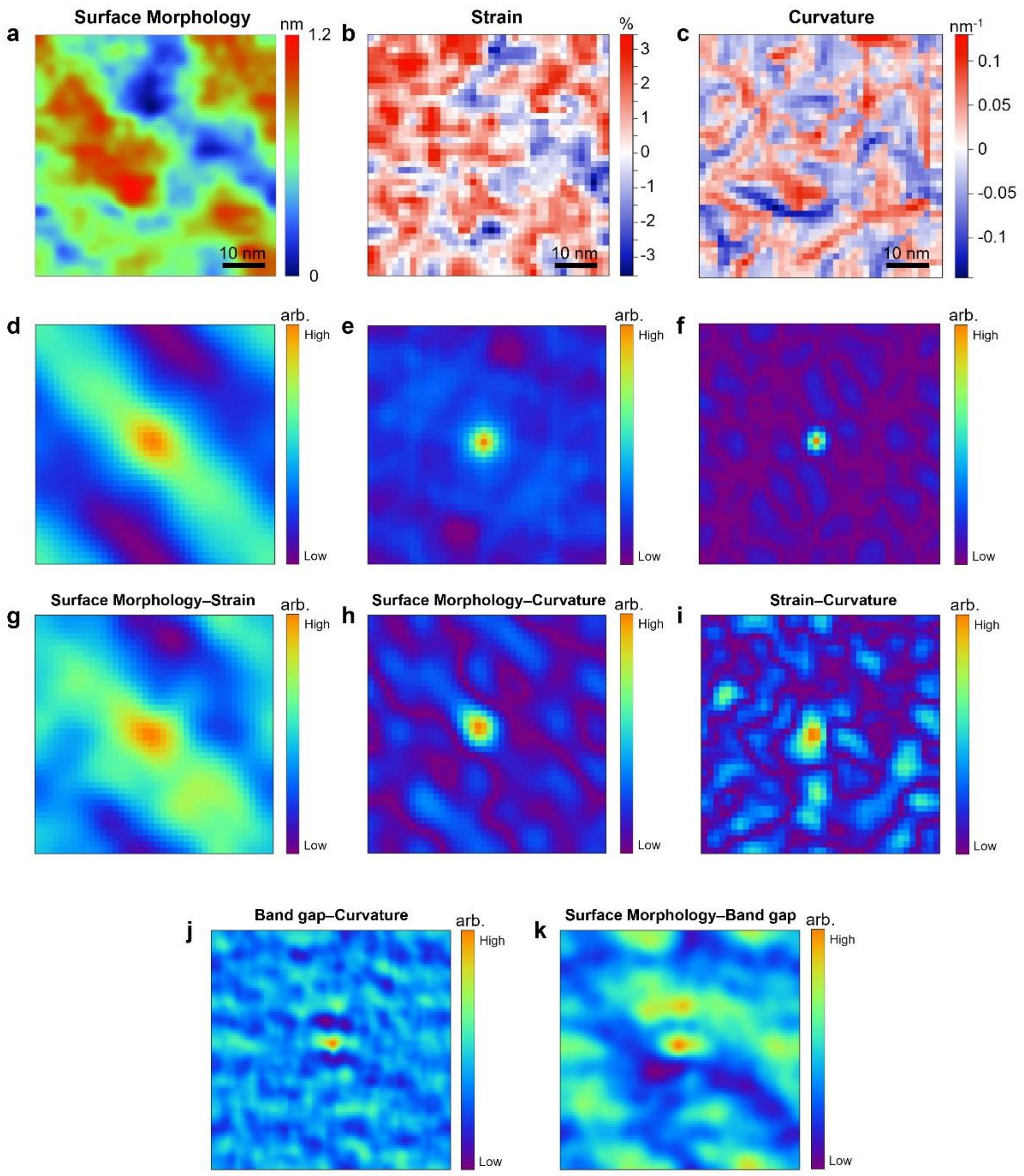

Supplementary Fig. 2 | Strain and curvature in monolayer MoS_2 on SiO_2 and correlations. a, Surface morphology of monolayer MoS_2 on SiO_2 in Supplementary Fig. 1a. **b**, Strain map of (**a**). Overall landscape with valleys and hills in surface morphology of (**a**) correlated with the strain distribution. **c**, Local absolute maximum of the principal curvatures with its sign, valley (−) and hill (+). **d**-**f**, Autocorrelation results of the surface morphology (**d**), strain (**e**), curvature (**f**) from (**a**-**c**). Autocorrelation for non-periodic images shows the trivial self-resemblance as a strong peak at the center. **g**-**i**, Cross-correlation results; (**g**) between surface morphology (**a**) and strain (**b**), (**h**) between surface morphology (**a**) and curvature (**c**), (**i**) between strain (**b**) and curvature (**c**). The strong peak at the center indicates a resemblance between the two quantities in comparison, which shows a high correlation. In particular, the cross-correlation between strain and curvature (**i**) shows the dominance of bending strain along with valleys and hills. The Poisson ratio of MoS_2 under

bending might cause the complexity of strain distribution[13], but the fluctuation of strain is still highly correlated with local curvature. **j**, Cross-correlation between band gap (Fig. 1c) and curvature (Fig. 1b). **k**, Cross-correlation between band gap and surface morphology (Fig. 1a). All the cross-correlation images show the largest central peak, indicating high correlation. The curvature correlated more sharply with the band gap as a main cause. The map of the local absolute maximum of the principal curvatures was similar to that of mean curvature, quantitatively. The Gaussian curvature did not show high correlation with the band gap.

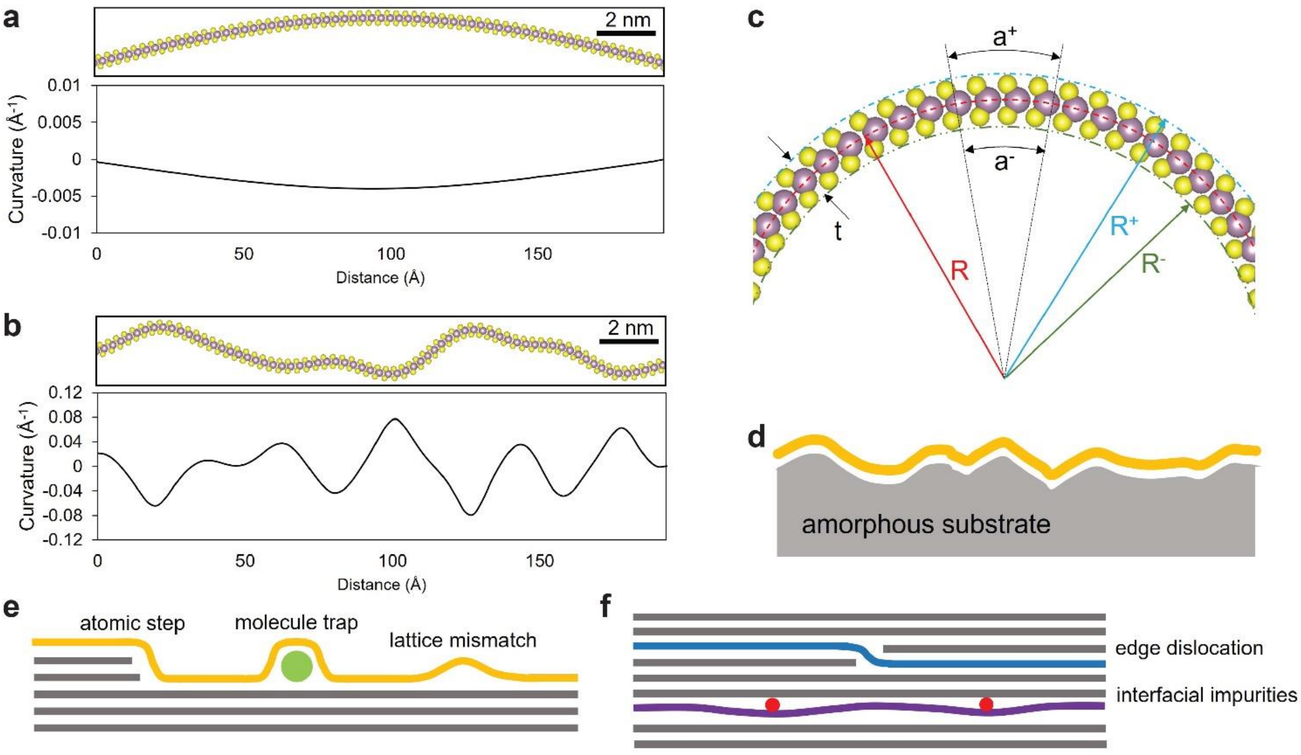

Supplementary Fig. 3 | Surface roughness, curvature, and bending strain. **a**, Height fluctuation of 1.6 nm with low values of slope changes (top inset), leading to a low curvature (bottom inset). **b**, In the same height fluctuation of (**a**) (top inset), the curvature is higher with larger values of slope changes (bottom inset), which indicates that the surface roughness should be characterized by not only root-mean-square but also slope changes. In the nanometer scale, curvature or bending strain can be significant even for atomic steps or atomic scale surface roughness[14,15]. **c**, Relation between curvature and bending strain. The outer surface of bent MoS_2 was observed by STM; if STM tip measures a negative (positive) curvature of a hill (valley) structure, the local lattice parameter ($a^{\pm}$) is larger (smaller) than the primitive lattice constant (a_0), i.e., STM tip measures $a^{+} \geq a_0$ for hills and $a^{-} \leq a_0$ for valleys. Simple bending strain ε follows that $\varepsilon = t/(2R)$, where t is the thickness of MoS_2 and R is the radius of curvature. The curvature is $1/R$. The measured surface was the outer surface in which the radius of curvature was obtained as R^{+} for hills and R^{-} for valleys from STM results. The simple bending strain can be obtained by that $\varepsilon = t/(2(R^{\pm} \mp t/2))$. **d-f,** Possible bending strain formations. (**d**) Surface roughness of an amorphous substrate. (**e**) Atomic steps in a crystalline substrate, interfacial molecule traps or lattice mismatch between 2D materials and a substrate. (**f**) Edge dislocation, interfacial impurities in 2D multilayers[16].

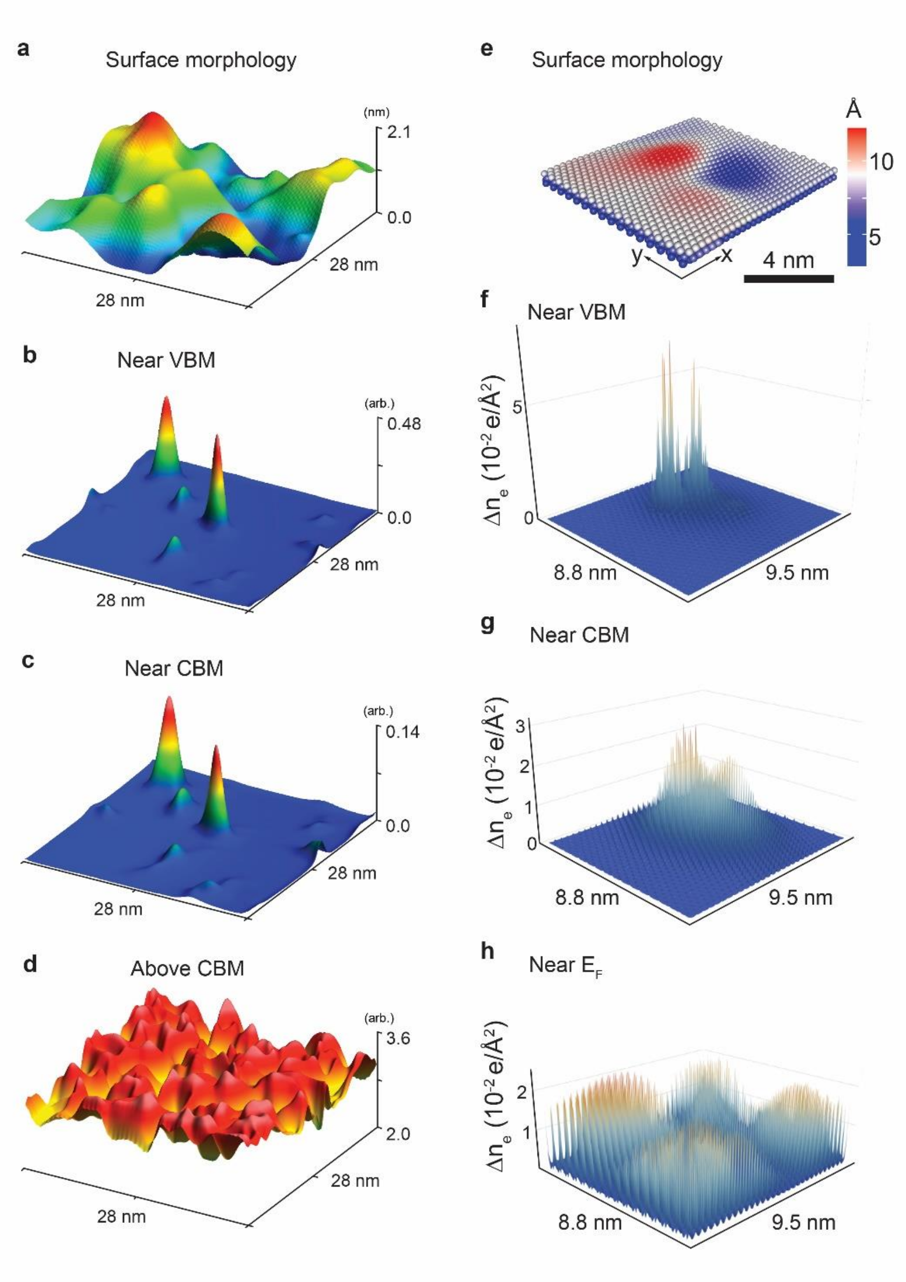

Supplementary Fig. 4 | Localized and extended states calculated by the tight-binding method and DFT in randomly deformed monolayer MoS_2. a, Calculated surface morphology for tight-binding method with the emphasized height axis. **b**, **c,** Localized states near the deep band tails of VBM (**b**) and CBM (**c**). **d**, Extended states above the energy of the CBM. Relative energies of (**b-d**) are given by −0.58, 0.58, and 1.16 eV, respectively. The Fermi level is set to zero. **e**, The structural model in Fig. 5c for DFT calculations was displayed in a different view in the original scale. When the curved structure was electron-doped (66 *e*/unit-cell ~ 7.95×10^{13} *e*/cm^2), the doping charge localization was exhibited near the band tails of the VBM (**b**) and the CBM (**c**). (**d**) The extended states were manifested near the Fermi level, which means that a metallic phase is expected at a high doping level. All the calculated results are in good agreement with the experimental results shown in Figs. 1d-f and 4a-d.

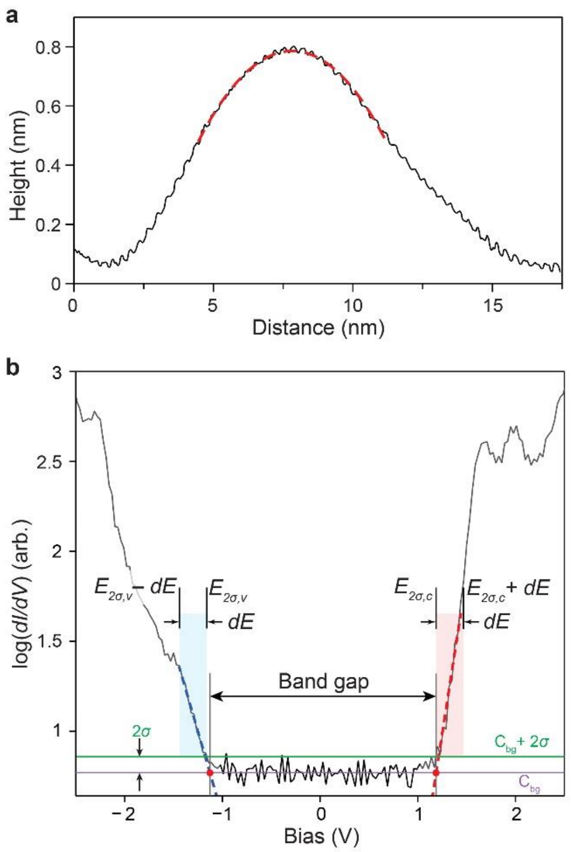

Supplementary Fig. 5 | Characterization of curvature and band gap. **a**, A typical line profile of deformed MoS_2 with a curvature. The dashed red line is a fitting result of the hill feature in the height change of MoS_2. The STM results show the isosurface of electron charge density related to the positional changes (of nuclei) and orbitals of each atom. The small corrugation of the picometer scale corresponds to the atomic protrusions of S atoms, which were not considered for the curvature analysis. **b**, Determination of band gap in STS spectra. The band edges (or band gap) were determined by linear fitting of log(dI/dV) (dashed lines) in the energy range of $E_{2\sigma,c(v)}$ to $E_{2\sigma,c(v)}$ + (−) dE, where $E_{2\sigma,c(v)}$ is the energy point of intersection between log(dI/dV) and a level that the average of the background noise (C_{bg}) plus twice the standard deviation of the background noise (2σ) in the band gap region (green line), and dE is 300 meV. The band edges were recorded at the intersections of the linear fits and C_{bg} (purple line). These are indicated by red dots on the plot. The blue and red shaded regions indicate the energy ranges (dE) for the linear fitting. This empirical determination procedure of band gap was suggested in the ref. 17.

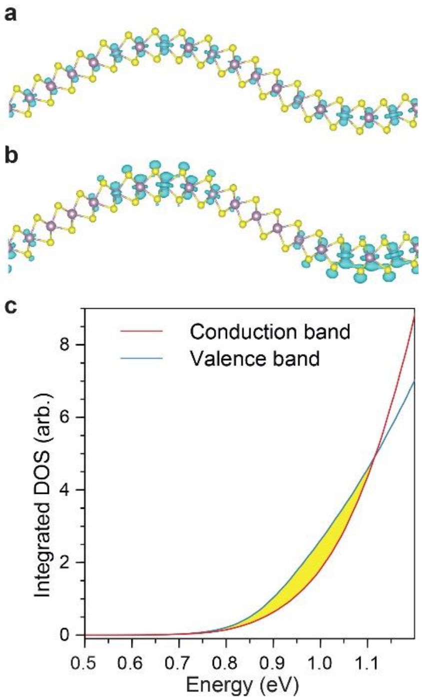

Supplementary Fig. 6 | Asymmetry of the density of states in deformed MoS_2. **a**, Doping charge density near the conduction band edge with the energy range of 0.3 eV. **b**, Doping charge density near the valence band edge with the energy range of 0.3 eV. The electron-doping concentration in (**a**) and (**b**) is 13.8×10^{13} e/cm^2. The isosurface of charge density in (**a**) and (**b**) is 10^{-4} e/Å^3. **c**, Integrated DOS near the band edges for the intrinsic case of the deformed MoS_2. The integration of DOS was performed from the Fermi level to each band side. The valence band edge shows a larger capacity than that of the conduction band edge. The yellow shade shows the difference between conduction and valence bands near the band edges. The energy is an absolute value. The zero energy is the Fermi level.

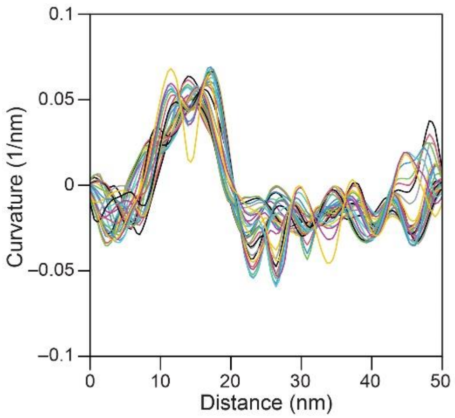

Supplementary Fig. 7 | Total curvature plot from Fig. 3a. Each line profile of curvature in Fig. 3a was plotted. The average value of the absolute curvatures was given in Fig. 3d.

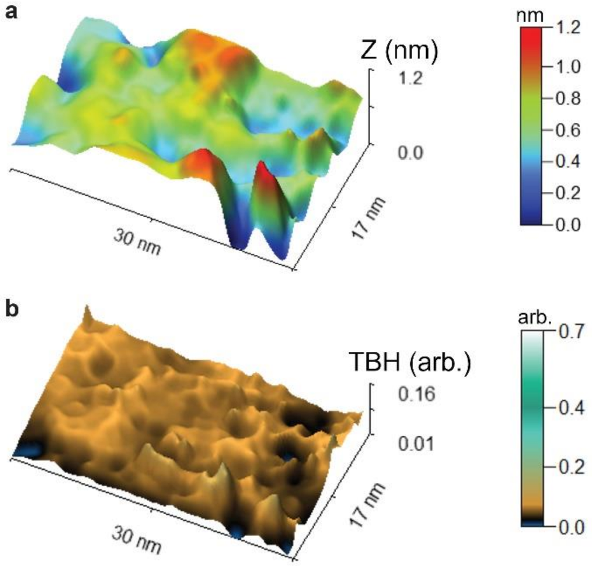

Supplementary Fig. 8 | Tunneling barrier height (TBH) map in the neutral monolayer MoS_2 on SiO_2. a, Surface morphology of the monolayer MoS_2 on SiO_2. **b**, Tunneling barrier height over the same area in (**a**) at the neutral state without applying a gate bias. The fluctuation of the tunneling barrier height is ~4 times smaller than those at gate bias of 70 V in Fig. 3b. The sample bias of −2.5 V was applied.

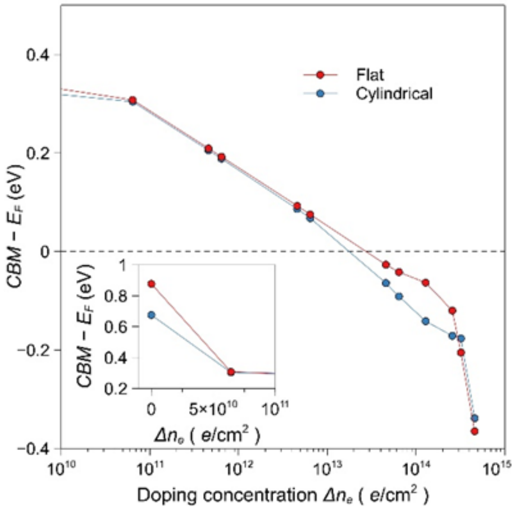

Supplementary Fig. 9 | DFT calculations of the relative energy position of the conduction band minimum (CBM) to the Fermi level as a function of doping. The Fermi level (E_F) changes continuously up to the high doping level of ~10^{15} *e*/cm^2. The difference in $CBM - E_F$ between the cylindrical curvature structure and the flat structure became significant after the E_F reached the CBM (i.e., $CBM – E_F = 0$) at the doping level above ~2×10^{13} *e*/cm^2. This is attributed to the band tails near the band edges, which are already occupied to reduce the effective capacity of doping charge above the conduction band edge. Therefore, the Fermi level change is enhanced in the curvature structure. Above the ~3×10^{14} *e*/cm^2, however, the $CBM - E_F$ of flat and cylindrical structures become similar again including the effect of the flattening of band edges which started to be saturated at ~5×10^{13} *e*/cm^2. The inset shows the neutral states ($\Delta n_e = 0$) of both cases. The smaller value of $CBM – E_F$ for the cylindrical case at the neutral state is due to the curvature-induced band gap fluctuation.

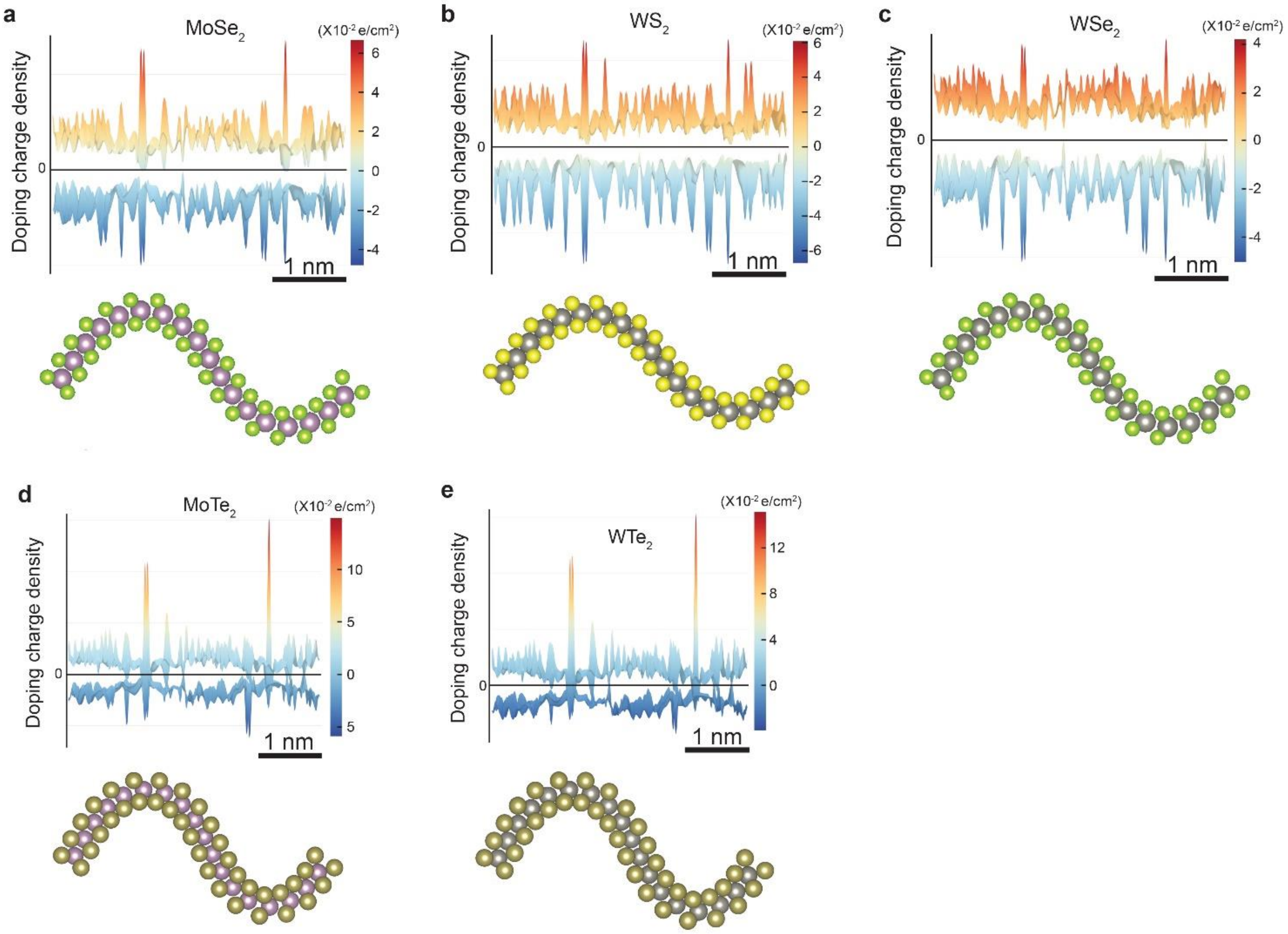

Supplementary Fig. 10 | Doping charge localization in cylindrical curvature structures in various transition metal dichalcogenides. a-e, The DFT-calculated doping charge densities in various transition metal dichalcogenides (MX_2, M = {Mo, W}, X = {S, Se, Te}) show doping charge localization in large curvature regions. The positive and negative doping charge densities reflect electron and hole doping, respectively. (**a**) $MoSe_2$, (**b**) WS_2, (**c**) WSe_2, (**d**) $MoTe_2$, and (**e**) WTe_2.

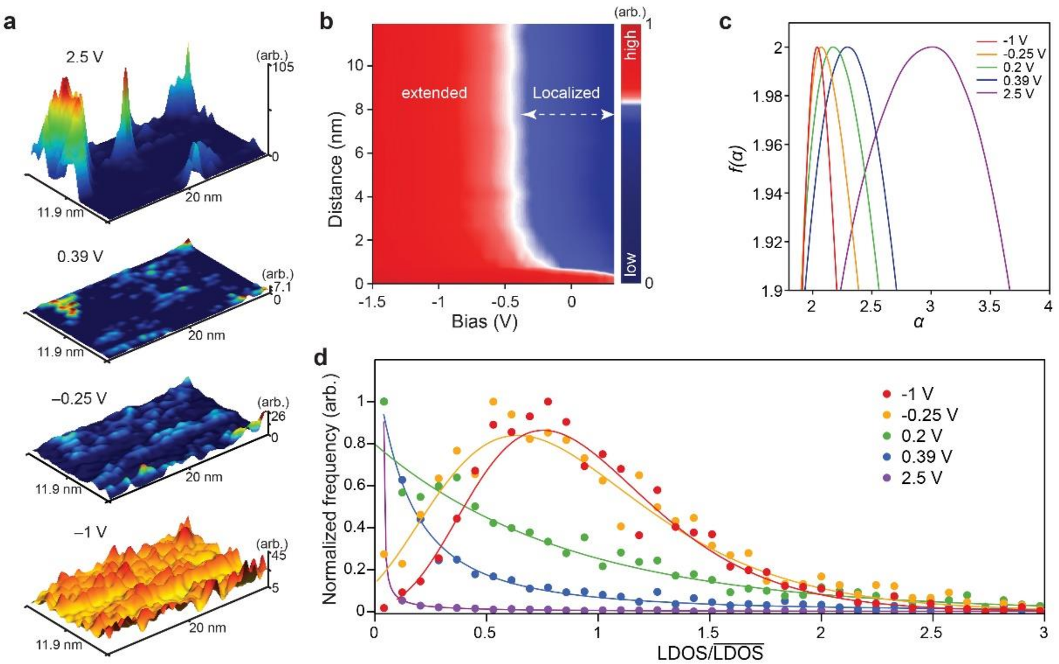

Supplementary Fig. 11 | Autocorrelation and multifractality in a hole-doping case. **a**, LDOS maps for localized states near VBM and CBM (sample bias: −0.25 to 2.5 V) and extended state (sample bias: −1 V) in the hole-doping case of Fig. 1m. **b**, Radial-averaged autocorrelation results of Fig. 1m. The Fermi level is set to zero. **c**, Singularity spectra for LDOS maps for the hole-doping case. The extended state (−1 V) is close to the metallic limit (the narrow region near $\alpha = 2$ satisfying with $f(\alpha = 2) = 2$) due to the flattening of the valence band edge involving with narrower band tail width than that of conduction band edge. The localized states (−0.25 to 2.5 V) exhibit stronger multifractality that the peak positions of singularity spectra are off-centered from $\alpha = 2$. **d**, Normalized LDOS distribution ($\mathrm{LDOS}/\overline{\mathrm{LDOS}}$) for the extended state (−1 V) shows a peak position near 1 of $\mathrm{LDOS}/\overline{\mathrm{LDOS}}$ indicating a uniform distribution. For the localized states, strong localization shows strongly skewed log-normal distributions of LDOS. If the hole-doping concentration becomes higher, the valence band edge will be more uniform exhibiting a narrower band tail width near the valence band edge and sharper LDOS distribution near its average value ($\mathrm{LDOS}/\overline{\mathrm{LDOS}} = 1$) following a normal distribution. In addition, the neutral (intrinsic) state of structural-disordered MoS_2 shows an even band tail width for the valence and conduction band edges by curvature formations, fast decaying behaviors in autocorrelation (short localization/correlation length), and strong multifractality due to the disorder-induced inhomogeneity of LDOS (Fig. 1d-f) without the flattening of band edges.

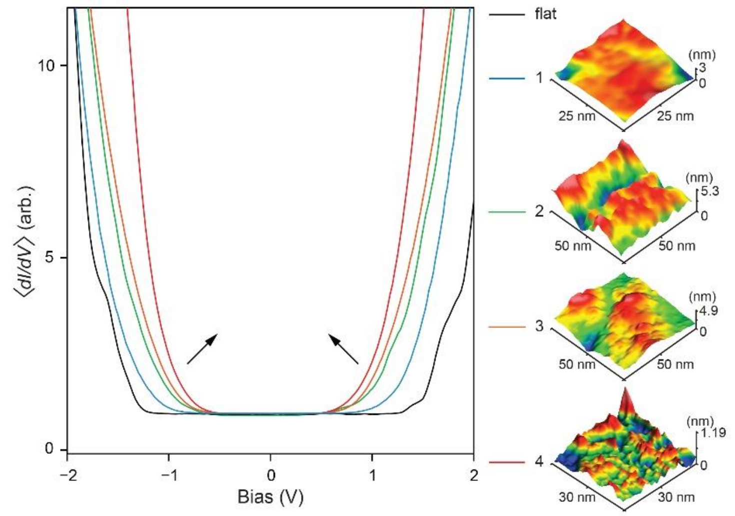

Supplementary Fig. 12 | Band tail formation in structural disorder. Rough MoS_2 surface with higher bending curvatures shows larger band tail width in comparison with the band tail in the flat region. <*dI/dV*> indicates the spatial average of *dI/dV* results corresponding to the density of states. The flat region (denoted as ‘flat’) shows a larger band gap with negligible band tail widths, which is close to the intrinsic property of monolayer MoS_2. The band tails of the regions labeled ‘1’ to ‘4’ in the side insets exhibit significantly different widths. The average values of mean curvatures in the ‘1’, ‘2’, ‘3’, and ‘4’ regions are 0.1593, 0.1763, 0.1867, and 0.1995 nm^{-1}, respectively. Each curvature value of ‘1’ to ‘4’ corresponds to the bending strain of 2.632, 2.912, 3.111, and 3.3589%, respectively. The higher value of curvature induces a more protruded band tail and larger band tail width, which is in good agreement with the theoretical calculations in Fig. 4i. The arrows indicate protrusions of band tails.

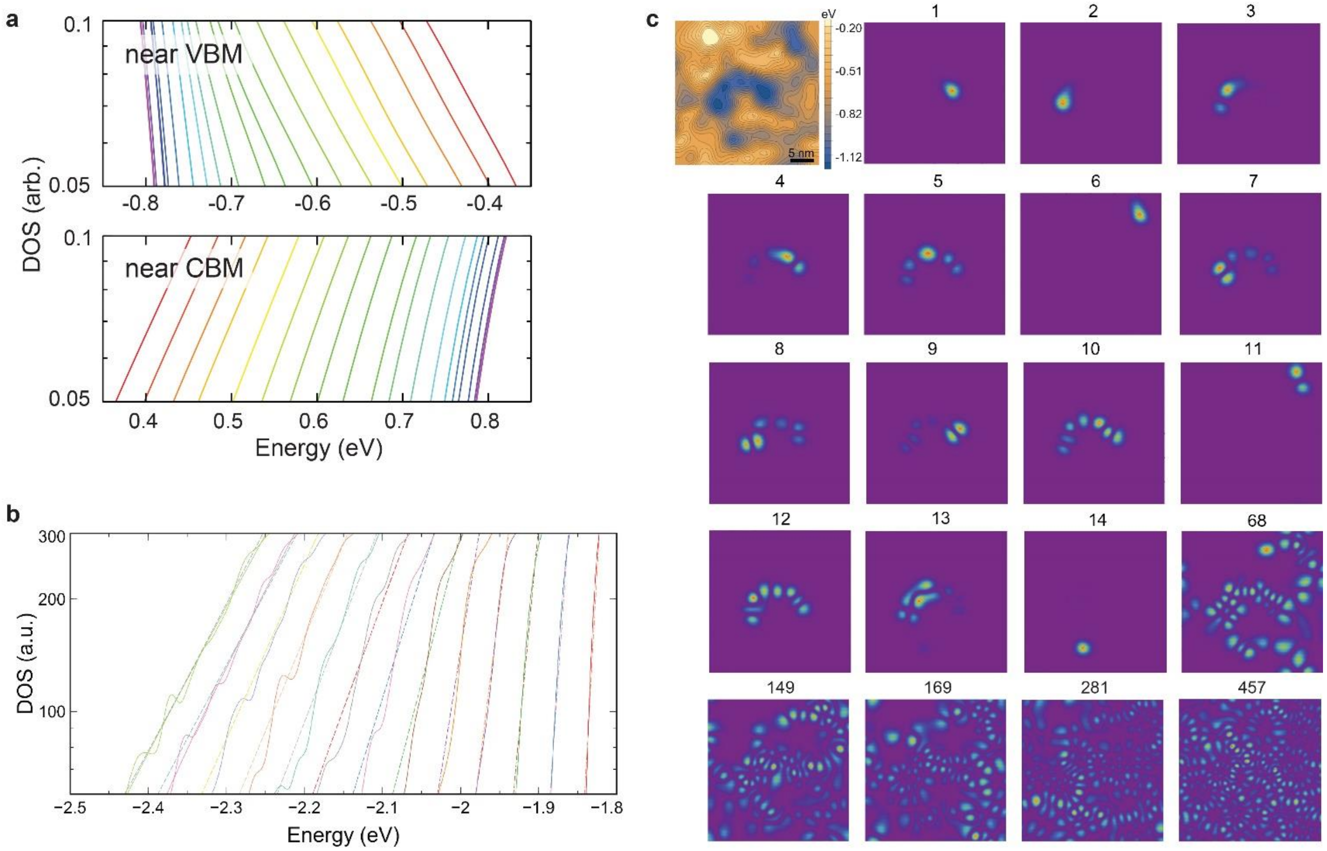

Supplementary Fig. 13 | Exponential band tails near the band edges. **a**, Close-up log-scale plots for Fig. 4i. (Top in (**a**)) Near the valence band edge. (Bottom in (**a**)) Near the conduction band edge. The linearity in the log-scale plots exhibits exponential behaviors. The color scale is the same in Fig. 4i. **b**, A Log-scale plot of exponential band tails for each scale of disorder strength calculated by the Schrödinger equation approach. The Schrodinger equation approach shows formations of the exponential band tails by each scaled random potential. Dashed lines are fitting results with exponential functions in each case. As the disorder strength for each result increases (from right to left in plot (**b**)), the exponential band tail width increases. The small fluctuation of DOS is due to the finite size effect of the model. c, Landscapes of a random potential (the first image at the top) and eigenstates (the numbered images). From the lowest energy level to the next 13 adjacent low energy levels, the states are strongly localized near the local minima of the random potential. In contrast to, the extended states at the higher energy levels (larger than 14) spread over the entire area.

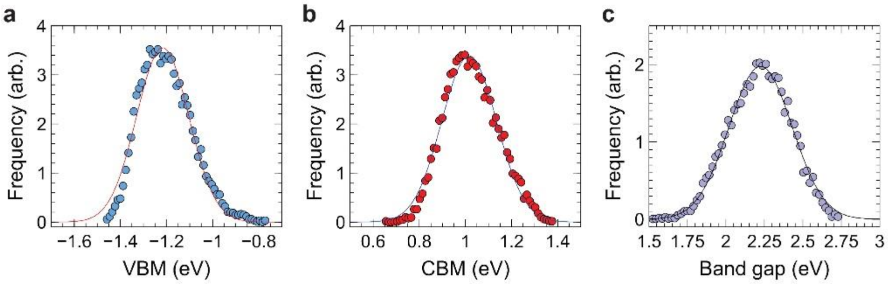

Supplementary Fig. 14 | Distributions of band edges and band gap in Fig. 1g. Distributions of (**a**) VBM, (**b**) CBM, and (**c**) band gap. A solid line of each is a fitted curve of the Gaussian function as a normal distribution.

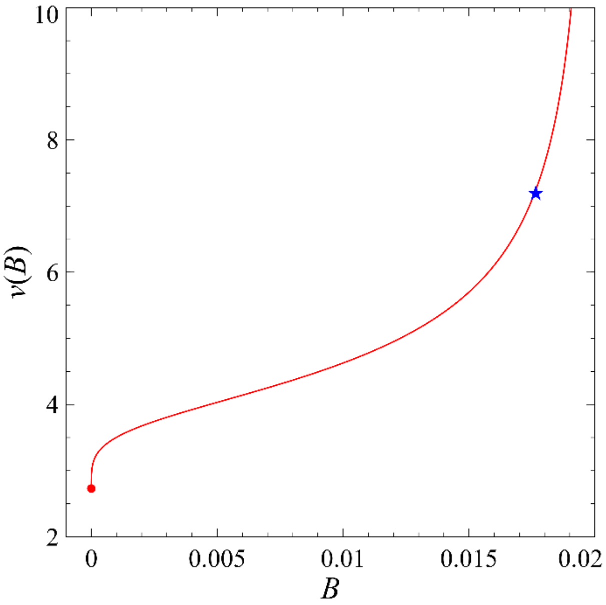

Supplementary Fig. 15 | The critical exponent ν in a 2D system with random spin-orbit coupling under weak magnetic field B (ref.[9]). The point at zero field indicates 2.73 as in a symplectic case. The magnetic field B in the eq. (9) is presented in the unit of the magnetic flux quantum, $h/|e|$ where h is the Planck constant and e is the electron charge. The averaged magnetic field of ~0.021 T from the Gaussian curvature of the STM result (related to Fig. 1j) corresponds to B = ~0.01765 $h/|e|$, which is denoted by a blue star ($\nu(B)$ = ~7.19) in the plot.

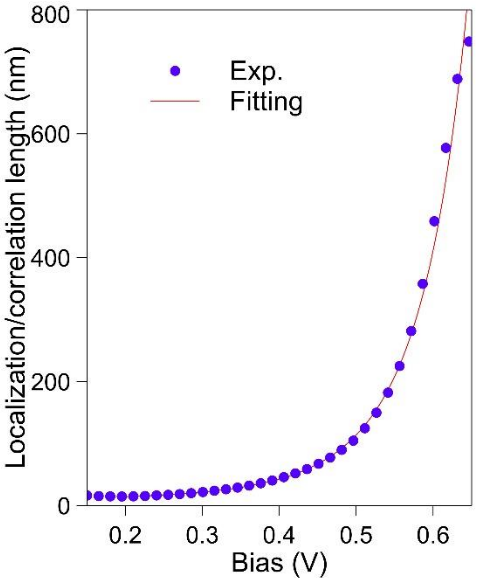

Supplementary Fig. 16 | Characterization of localization/correlation length near the critical energy. The radial-averaged autocorrelation profiles (Fig. 4e and See Methods.) were characterized by fitting the localization/correlation length of the equation, $\sim\exp(-|\boldsymbol{R}|/\xi)$ where $|\boldsymbol{R}|$ is the radial distance and ξ is the localization/correlation length. To obtain the critical exponent ν from the localization/correlation lengths from the above, the equation of $\sim|E - E_{C,}|^{-\nu}$ was fitted. The obtained critical exponent value is 2.73 which is consistent with the contour fitting results of the radial-averaged autocorrelation profile (Fig. 4e) and the theoretical values of the curvature-induced pseudo-magnetic field (Supplementary Fig. 15) as described in the main manuscript. All the results from independent approaches agreed well with each other, suggesting that the curvature mechanism of electronic/spin structure played an important role.

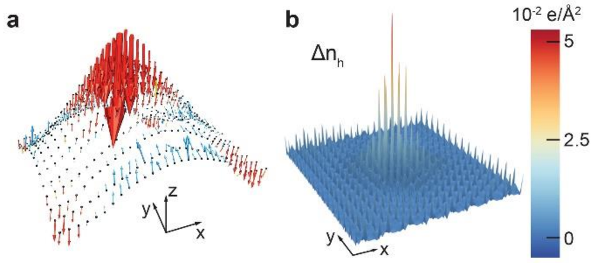

Supplementary Fig. 17 | Correlation between magnetic moment and hole-doping charge density in a spherical curvature. **a,** Localized magnetic moment in the spherical curvature structure of monolayer MoS_2 (as the inset in Fig. 5a). Dots indicate the surface of the structure. The red and blue colors of the arrows correspond to the downward and upward directions of the magnetic moments, respectively. The sizes of the arrows are proportional to the magnitudes of local magnetic moments. **b**, Hole-doping charge density is localized at the center of the spherical curvature. The hole doping level is $-4e$/unit-cell. The magnitude of magnetic moments correlated with the charge density in the spherical curvature.

Supplementary References

1. Boettcher, S. W., Oener, S. Z., Lonergan, M. C., Surendranath, Y., Ardo, S., Brozek, C. & Kempler, P. A., Potentially confusing: potentials in electrochemistry. *ACS Energy Lett.* **6**, 261 (2021).
2. Evers, F. & Mirlin, A. D., Anderson transitions. *Rev. Mod. Phys.* 80, 1355 (2008).
3. Ferrari, L. & Russo, G., Urbach tails in chalcogenides: a self-consistent approach. *Phil. Mag. B* **63**, 501-511 (1991).
4. Chen, X., Wu, Z., Xu, S., Wang, L., Huang, R., Han, Y., Ye, W., Xiong, W., Han, T., Long, G., Wang, Y., He, Y., Cai, Y., Sheng, P. & Wang, N., Probing the electron states and metal-insulator transition mechanisms in molybdenum disulphide vertical heterostructures. *Nat. Commun.* **6**, 6088 (2015).
5. Cohen, M. H., Chou, M. Y., Economou, E. N., John, S. & Soukoulis, C. M., Band tails, path integrals, instantons, polarons, and all that. *IBM J. Res. & Dev.* **32**, 82-89 (1988).
6. Bacalis, N., Economou, E. N. & Cohen, M. H., Simple derivation of exponential tails in the density of states. *Phys. Rev. B* **37**, 2714 (1988).
7. Ochoa, H., Zarzuela, R. & Tserkovnyak, Y., Emergent gauge field from curvature in single layers of transition-metal dichalcogenides. *Phys. Rev. Lett.* **118**, 026801 (2017).
8. Gutiérrez-Rubio, A., Stauber, T., Géomez-Santos, G., Asgari, R. & Guinea, F., Orbital magnetic susceptibility of graphene and MoS_2. *Phys. Rev. B* **93**, 085133 (2016).
9. Su, Y., Wang, C., Avishai, Y., Meir, Y. & Wang, X. R., Absence of localization in disordered two-dimensional electron gas at weak magnetic field and strong spin-orbit coupling. *Sci. Rep.* **6**, 33304 (2016).
10. Li, H., Zhang, Q., Yap, C. C. R., Tay, B. K., Edwin, T. H. T., Olivier, A. & Baillargeat, D., From Bulk to Monolayer MoS_2: Evolution of Raman Scattering. *Adv. Funct. Mater.* **22**, 1385 (2012).
11. Splendiani, A., Sun, L., Zhang, Y., Li, T., Kim, J., Chim, C.-Y., Galli, G. & Wang, F., Emerging Photoluminescence in Monolayer MoS_2. *Nano Lett.* **10**, 1271 (2010).
12. Mouri, S., Miyauchi, Y. & Matsuda, K., Tunable Photoluminescence of Monolayer MoS_2 via Chemical Doping. *Nano Lett.* **13**, 5944 (2013).
13. Liu, X., Pan, D., Hong, Y. & Guo, W., Bending Poisson effect in two-dimensional crystals. *Phys. Rev. Lett.* **112**, 205502 (2014).
14. Rokni, H. & Lu, W., Direct measurements of interfacial adhesion in 2D materials and van der Waals heterostructures in ambient air. *Nat. Commun.* **11**, 5607 (2020).
15. Yu, J., Han, E., Hossain, M. A., Watanabe, K., Taniguchi, T., Ertekin, E., van der Zande, A. M. & Huang, P. Y., Designing the bending stiffness of 2D material heterostructures. *Adv. Mater.* **33**, 2007269 (2021).
16. Rooney, A. P., Kozikov, A., Rudenko, A. N., Prestat, E., Hamer, M. J., Withers, F., Cao, Y., Novoselov, K. S., Katsnelson, M. I., Gorbachev, R. & Haigh, S. J., Observing imperfection in atomic interfaces for van der Waals heterostructures. *Nano Lett.* **17**, 5222 (2017).

17. Ugeda, M.M., Bradley, A. J., Shi, S.-F., da Jornada, F. H., Zhang, Y., Qiu, D. Y., Ruan, W., Mo, S.-K., Hussain, Z., Shen, Z.-X., Wang, F., Louie, S. G., Crommie, M. F., Giant bandgap renormalization and excitonic effects in a monolayer transition metal dichalcogenide semiconductor, *Nat. Mater.* **13**, 1091 (2014).
